# The Dangerous Drugs Hospital Scheme

**Published:** 1921-06-11

**Authors:** F. A. Hocking

**Affiliations:** London Hospital, Whitechapel Road, E. 1


### THE EDITOR'S LETTER-BOX.

**Correspondence on all subjects is invited, but we cannot in any way be responsible for the opinions expressed by our correspondents, who must give their name and address as a guarantee of good faith, but not necessarily for publication. Correspondents are reminded that brevity of style and conciseness of statement greatly facilitate early insertion.**

### The Dangerous Drugs Hospital Scheme.

*To the Editor of* The Hospital.

Sir,—With reference to your note on page 164 of The Hospital, concerning "the clause allowing the ward sister to obtain on her own requisition supplies of morphia injection, etc.," may I be permitted to point out that this is not exactly what the Home Office scheme states. The official clause is : "Supplies of stock preparations will only be *replenished* on a written requisition from the sister-in-charge." This clause does not confer upon the ward sister any right to determine what drugs or preparations are to be kept as stock in the ward medicine cupboards. The responsibility for deciding what shall or shall not be "stock" rests where it has always rested—*viz.*, with the competent authority of each institution. All that is permitted to a ward sister is the maintenance of sufficient supplies of stock preparations so long as such preparations are permitted to be *stock* by the competent authority. In practice her requisitions are subject to the discretion of the hospital pharmacist.—Yours faithfully,

F. A. Hocking.

London Hospital, Whitechapel Road, E. 1,

June 4, 1921.

[On inquiry we find that at many of the medium-sized hospitals, and at least one large hospital in London, supplies of morphia, etc., are only "replenished" when the requisition is signed by a medical officer of the institution. The fact remains that if (as is customary and necessary) morphia injection is on the list of preparations to be kept in stock in the ward, supplies may now be obtained (replenished) by the ward sister on her own requisition. Mr. Hocking says "her requisitions are subject to the discretion of the hospital pharmacist." We are not convinced that this official is always in a position to decide, as he has no record furnished to him of the precise manner in which the last supply has been used. If, however, orders to replenish were signed by a medical officer, the pharmacist would execute the order and his responsibility ceases. The medical officer, being responsible, is likely to satisfy himself that supplies are not replenished more frequently than should be necessary to fulfil his orders. In the case of prescriptions for individuals it is to be noted that the hospital scheme demands that if a supply of medicine coming under the Act is to be replenished the supply may only be issued on the direct order of a medical practitioner. No discretionary power is here vested in the pharmacist. The Pharmaceutical Society's scheme proposed that the clause in question should read : "Supplies . . . on written requisition of the medical officer *or* sister-in-charge of the ward, according to the custom of the institution." To our mind this would have been preferable, allowing the many institutions to continue the practice of recognising only the authority of a medical officer's order, whilst leaving such hospitals as desired it to continue to allow the ward sister to replenish stock on her own requisition. We are inclined to think that medical superintendents of hospitals who have up to now signed all requisitions to replenish ward stocks of morphia, etc., will not agree to delegate this duty to a ward sister. Perhaps we may have some comment from an institutional medical superintendent ?—The Writer of the Article.]

### Mr. Keyser's Proposal.

*To the Editor of* The Hospital.

